# A High Performance Banknote Recognition System Based on a One-Dimensional Visible Light Line Sensor

**DOI:** 10.3390/s150614093

**Published:** 2015-06-15

**Authors:** Young Ho Park, Seung Yong Kwon, Tuyen Danh Pham, Kang Ryoung Park, Dae Sik Jeong, Sungsoo Yoon

**Affiliations:** 1 Division of Electronics and Electrical Engineering, Dongguk University, 26 Pil-dong 3-ga, Jung-gu, Seoul 100-715, Korea; E-Mails: fdsarew@hanafos.com (Y.H.P.); sbaru07@dgu.edu (S.Y.K.); phamdanhtuyen@dongguk.edu (T.D.P.); jungsoft97@dongguk.edu (D.S.J.); 2 Kisan Electronics, Sungsoo 2-ga 3-dong, Sungdong-gu, Seoul 133-831, Korea; E-Mail: ssyoon@kisane.com

**Keywords:** banknote recognition, one-dimensional (line) sensor, pre-classification, USD banknote

## Abstract

An algorithm for recognizing banknotes is required in many fields, such as banknote-counting machines and automatic teller machines (ATM). Due to the size and cost limitations of banknote-counting machines and ATMs, the banknote image is usually captured by a one-dimensional (line) sensor instead of a conventional two-dimensional (area) sensor. Because the banknote image is captured by the line sensor while it is moved at fast speed through the rollers inside the banknote-counting machine or ATM, misalignment, geometric distortion, and non-uniform illumination of the captured images frequently occur, which degrades the banknote recognition accuracy. To overcome these problems, we propose a new method for recognizing banknotes. The experimental results using two-fold cross-validation for 61,240 United States dollar (USD) images show that the pre-classification error rate is 0%, and the average error rate for the final recognition of the USD banknotes is 0.114%.

## 1. Introduction

Functionality for detecting counterfeit banknotes and recognizing banknotes are required in various machines, such as banknote-counting machines and automatic teller machines (ATMs). Banknote recognition is defined as the recognition of the type of banknote (e.g., $1, $2, and $5), the direction of the banknote, and the date of issue of the banknote (e.g., least recent, recent, and most recent). Furthermore, banknote recognition facilitates the detection of counterfeit banknotes and allows the condition of banknotes to be monitored. Thus, research on banknote recognition has developed rapidly [1,2,3,4,5,6,7,8].

In general, a banknote’s obverse and reverse image differ. Hence, a banknote is constituted by four different patterns (obverse-forward, obverse-backward, reverse-forward, and reverse-backward) according to the direction of the banknote received by the machine. This increases the number of banknote classes four-fold. Therefore, the complexity of banknote recognition increases accordingly, which, in turn, increases the processing time and the banknote recognition error rate.

Previous studies can be divided into those that address the recognition of a banknote’s orientation [9] and those concerned with banknote recognition [10,11,12,13,14,15,16,17]. Wu *et al*. proposed a banknote-orientation recognition method using a back-propagation (BP) network [9]. They classified the input direction of banknotes by using a three-layer BP network. The performance of their method is high, however, they used only one type of banknote—viz., renminbi (RMB) 100 Yuan—in their experiment.

Kagehiro *et al*. proposed a hierarchical classification method for United States Dollar (USD) banknotes. Their method consists of three stages, using a generalized learning-vector quantization (GLVQ) algorithm to achieve high-speed processing with a high degree of accuracy [10]. The performance of their method was such that 99% of the banknotes were correctly recognized. Hasanuzzaman *et al*. proposed a banknote-recognition method based on speeded-up robust features (SURF) features [11]. Because it uses SURF features, their method is robust to illumination and scaling changes, as well as image rotation. At two seconds, however, the processing time required for a 3 GHz CPU computer is excessively long. Gai *et al*. proposed a feature-extraction method based on the quaternion wavelet transform (QWT) and generalized Gaussian distribution (GGD) for banknote classification [12]. With their method, USD banknotes are classified using a BP neural network. Ahmadi *et al*. and Omatu *et al*. proposed a banknote-classification method using principal component analysis (PCA), self-organizing map (SOM) clustering, and a learning-vector quantization (LVQ) classifier [13,14,15,16,17]. They presented a method to improve the reliability of banknote classification using local PCA [13,14,15,16,17] for data-feature extraction.

In other research [18], Yeh *et al*. proposed a method for detecting counterfeit banknotes based on multiple-kernel support vector machines (SVMs). They segmented a banknote into partitions, and took the luminance histograms of the partitions (with its own kernels) as the inputs to the system. In order to fuse the multiple kernels, they used semi-definite programming (SDP) learning. Unlike our research, their method is not concerned with recognizing the type of USD banknote. Rather, it is designed to detect counterfeit Taiwanese banknotes. This is the central difference between their research and ours. Two classes are needed with their method [18]: genuine and counterfeit banknotes. Our research, however, requires 64 classes: four directions and 16 banknote types ($1, $2, $5, recent $5, most recent $5, $10, recent $10, most recent $10, $20, recent $20, most recent $20, $50, recent $50, most recent $50, $100, and recent $100).

In previous research [19,20], Bruna *et al*. proposed a method to detect various types of counterfeit Euro banknotes. They used a near-infrared (NIR) light illuminator and a camera to capture the banknote image. This differs from our method, which uses a visible-light USD image. In order to discriminate counterfeit from genuine banknotes, they used a percentage of the pixels that satisfy predetermined conditions inside predefined regions. In addition, they used a correlation measure between the patches learned at the training stage and the corresponding pixels in the searching regions to recognize of the banknote type.

Hasanuzzaman *et al*. proposed a method for recognizing banknotes for the visually impaired based on SURF and the spatial relationship of matched SURF features [21]. Although they obtained a correct-recognition rate of 100% with USD banknotes, the number of classes and images for testing, at seven and 140, respectively, was too small. As noted above, by contrast, our experiments involved 64 classes and 61,240 images, as shown in Table 2.

In previous studies on banknote recognition, each banknote type was considered as constituted by four classes (obverse-forward, obverse-backward, reverse-forward, and reverse-backward), rather than a single class. Consequently, the number of banknote classes is considerably large. To overcome these problems, we propose a new method for recognizing banknotes. When compared with previous studies, the proposed method is novel in the following four ways:

(1) The region-of-interest (ROI) area on the captured banknote image is located by corner detection algorithm, but there still exist the effect of the misalignment, geometric distortion, and non-uniform illumination on the ROI image. Therefore, a sub-sampled image of 32 × 6 pixels was used in our research in order to extract features more efficiently by reducing this effect.
(2) In order to further reduce this effect on the recognition accuracy, we propose the feature extraction method by PCA with the sub-sampled image.
(3) Pre-classification is performed hierarchically: the first classification demarcates the obverse and reverse side, and the second demarcates the forward and backward direction. This pre-classification process reduces classification errors.
(4) Pre-classification is performed using a SVM based on the optimal feature vector extracted using PCA on the sub-sampled image. Then, the final type of banknote is recognized using the classifier based on a K-means algorithm.

Table 1 compares the methods proposed in previous studies with ours. The remainder of our paper is organized as follows: we describe the proposed method in Section 2. In addition, we present experimental results, discussions, and concluding remarks in Section 3, Section 4 and Section 5, respectively.

## 2. Proposed Method

### 2.1. Overall Procedure

**Figure 1:**
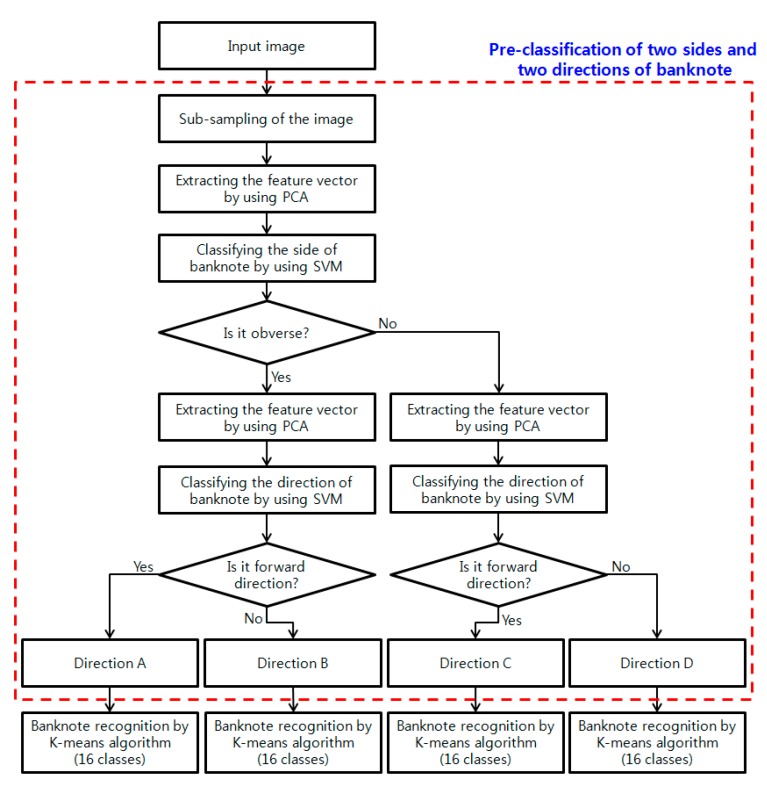
Overview of the proposed method.

An overview of our pre-classification method for the recognition of USD banknotes is provided in Figure 1. We defined four directions in our study: the obverse side in a forward direction is “Direction A”, the obverse side in a backward direction is “Direction B”, the reverse side in a forward direction is “Direction C”, and the reverse side in a backward direction is “Direction D”.

**Table 1:** Comparison of the proposed method with previous methods.

Category	Methods	Strengths	Weakness
Non-pre-classification-based method	-Using GLVQ [10]	Additional processing time for pre-classification is not required	Accuracy enhancement is limited because of the large number of classes of banknotes, including two sides (obverse and reverse) and two directions (forward and backward)
	-Using SURF features [11]		
	-Using QWT, generalized Gaussian distribution, and BP neural network [12]		
	-Using local PCA, SOM, and LVQ [13,14,15,16,17]		
	-Using correlation measure [19,20]		
	-Using SURF and the spatial relationship of matched SURF features [21]		
Pre-classification-based method	-Using BP neural network [9]	The number of classes of banknotes can be reduced four-fold, because of the pre-classification of the two sides and two directions	The classification accuracy of banknote type is not presented
	-Using SVM classifier with PCA features **(proposed method)**		Additional processing time is required for pre-classification

As shown in the figure, our method consists of four steps. First, to reduce the processing time, the input image is sub-sampled to yield a single image that is 32 × 6 pixels in size. Second, the optimal feature vector is extracted using PCA to classify the side of the banknote. Third, the side of the input image is determined as either obverse or reverse using an SVM. Fourth, the optimal feature vector for classifying the banknote’s direction is extracted using PCA. Finally, the forward or backward direction is determined using the SVM. Then, the final kind of banknote is determined by K-means algorithm of 16 classes using the PCA features of the sub-sampled image.

### 2.2. Image Acquisition and Pre-Processing

In this study, the banknote image was captured using a commercial banknote-counting machine. Due to the size and cost limitation of the banknote-counting machine, the banknote image of visible light is captured by one-dimensional (line) sensor instead of conventional two-dimensional (area) sensor. That is, one line (row) image is acquired instead of a space (row by column) image at each time. The line image is acquired with visible light emitting diode (LED) while the input banknote is moving through the roller inside the banknote-counting machine at fast speed. The resolution of one line image is 1584 pixels, and 464 line images are acquired by this system. Based on this, the two-dimensional 1584 × 464 pixels image of the banknote is finally acquired by sequentially combining the 464 line images.

**Figure 2:**
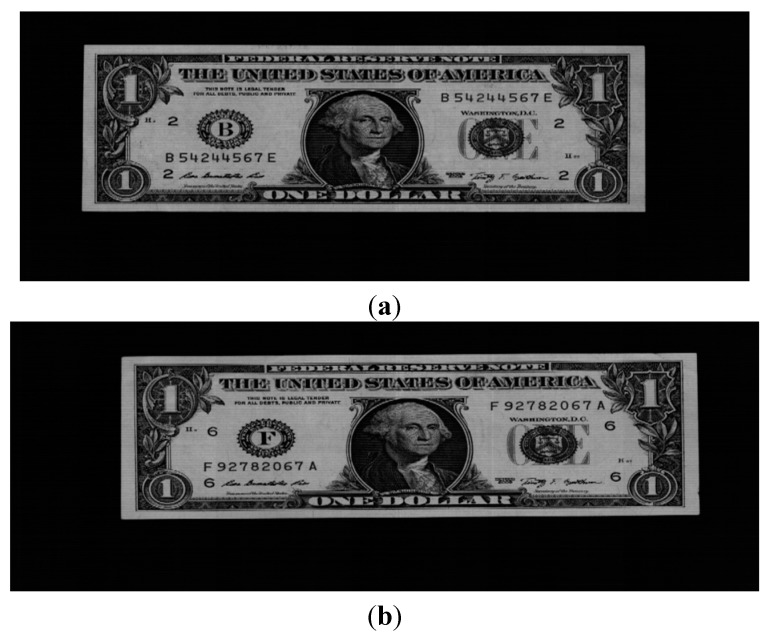

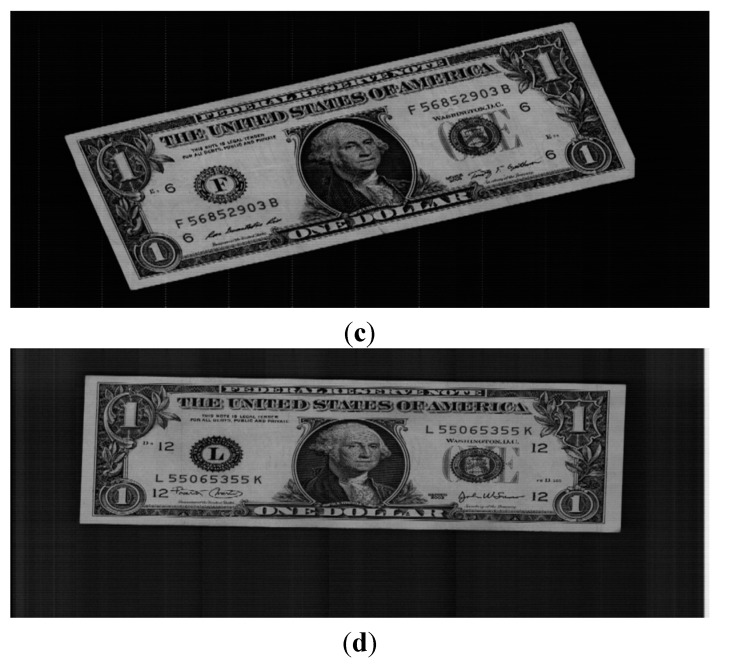
Examples of misalignment, geometric distortion, and non-uniform illumination on the captured images: the cases of (**a**); (**b**) misalignment; (**c**) geometric distortion; and (**d**) non-uniform illumination.

Because the banknote image is captured by the line sensor while it is moved through the roller at fast speed misalignment, geometric distortion, and non-uniform illumination effects frequently occur in the captured images, as shown in Figure 2, and the images with these effects are included in our database for experiments.

**Figure 3:**
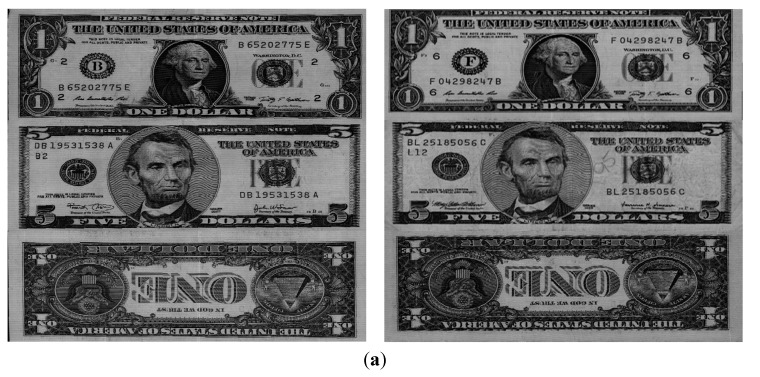

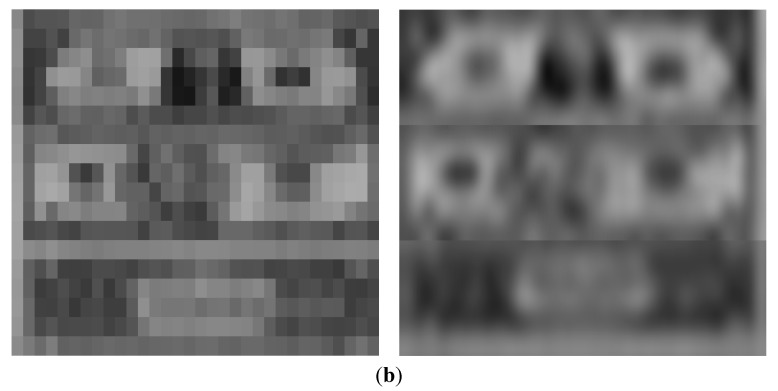
Examples of sub-sampled images: (**a**) original ROI areas of the banknote; (**b**) sub-sampled images.

To overcome these problems, the ROI area on the banknote is located by corner detection algorithm, but there still exist the positional variations on the ROI image as shown in Figure 3. In addition, the image resolution for the ROI area on banknote is as high as 1212 × 246 pixels, which leads to considerable processing time and the inclusion of noise and redundant data. Therefore, a sub-sampled image of 32 × 6 pixels was used in our research in order to extract features more efficiently by reducing the processing time and any unnecessary data without the positional variations, as shown in Figure 3.

The images of the 1st row of Figure 3b are the sub-sampled ones of the 1st row of Figure 3a. Like this, the images of the 2nd, and 3rd rows of Figure 3b are the sub-sampled ones of the 2nd, and 3rd rows of Figure 3a, respectively. If we show the sub-sampled image as its actual size (32 × 6 pixels), it is difficult for readers to discriminate the image because the size of sub-sampled image is much smaller than that (1212 × 246 pixels) of original ROI area of Figure 3a. Therefore, we show the magnified sub-sampled images whose size is larger than actual one (32 × 6 pixels) for higher visibility to readers in Figure 3b. Even with the sub-sampled image, there still exist the positional variations as shown in Figure 3b. Therefore, we propose the feature extraction method by PCA in order to reduce this effect on the recognition performance, and detail explanations are shown in the next section.

### 2.3. Feature Extraction by PCA, and Classification with an SVM

In general, as the dimensionality of data increases, feature extraction and pattern classification require much processing. In addition, an increase in the dimensionality can degrade the classification accuracy. PCA is a popular stochastic method that facilitates the analysis of high-dimensional data by dimensionality reduction [13,22]. This characteristic in PCA allows it to analyze the patterns of data more easily by reducing the dimensions of the data with minimal data loss. The procedure for conducting PCA is as follows. First, the covariance matrix Σ of the data is calculated:

(1) $$\text{Σ}=\frac{1}{\text{N}}\overset{\text{N}}{\underset{\text{n}=1}{\sum }}\left(x_{n}-\mu \right){\left(x_{n}-\mu \right)}^{\text{T}}$$

where N is the amount of data, *μ* is the mean of *x_n_*, and *x_n_* is the input data. Then, the eigenvalues and eigenvectors for the covariance matrix are calculated. In this study, the data represented by PCA is used as a feature vector for the classification of the banknote’s direction (“Direction A”, “Direction B”, “Direction C” and “Direction D” in Figure 1).

To classify the direction of the banknote, we used an SVM. In general, the decision function of an SVM is defined as [22,23]:

(2) $$f\left(x\right)=\text{sgn}\left(\overset{l}{\underset{1}{\sum }}y_{i}\alpha_{i}K\left(x,x_{i}\right)+b\right)\;$$

where *l* is the amount of data and $y_{i}\in \left[1,\;-1\right]$ is the indicator vector. In our study, the value “1” is assigned to the correct class and the value “−1” to the incorrect class. $\alpha_{i}$ and b are the weight value to $K\left(x,x_{i}\right)$ and off-set used in the decision function of SVM, respectively [22,23]. $K\left(x,x_{i}\right)$ is a kernel function. In our experiments, LibSVM software [24] was used to determine the optimal parameters. This software provides various kernel functions. Using training data to experiment, the linear kernel was selected as the optimal kernel for classifying the banknote’s direction. The classification step using the SVM classifier consists of two sub-steps, as shown in Figure 1. The first sub-step involves classifying the group of obverse sides (Direction A or B) and the group of reverse sides (Direction C or D). Finally, the direction of the banknote is determined in the second sub-step. For example, if the result of the first sub-step is that the banknote is determined to belong in the obverse-sided group, the direction of the banknote is determined as either Direction A or B in the second sub-step, as shown in Figure 1.

## 3. Experimental Results

We collected a database consisting of 61,240 USD banknote images for our experiments. The images in the database consisted of the four directions for the 16 types of banknotes ($1, $2, $5, recent $5, most recent $5, $10, recent $10, most recent $10, $20, recent $20, most recent $20, $50, recent $50, most recent $50, $100, recent $100). As shown in Table 2, the number of images used in our study is similar to that of [10], but much larger than in most previous studies [11,12,13,14,15,16,17]. In addition, our study uses more classes than any previous study [10,11,12,13,14,15,16,17]. Because there is no open database of USD banknote images, it is difficult to compare the performance of our method with those of previous researches on same condition. Therefore, we show that the number and classes of banknote images in our experiment are comparatively larger than those of previous researches in Table 2.

**Table 2:** Comparison of the number of images and classes used in previous studies with that of our study.

Method	The Number of Images	The Number of Classes (Including Two Sides and Two Directions)
[10]	65,700	48
[11]	140	28
[12]	15,000	24
[13,16]	2400	24
[14,15]	3600	24
[17]	3570	24
Our study	61,240	64

**Figure 4:**
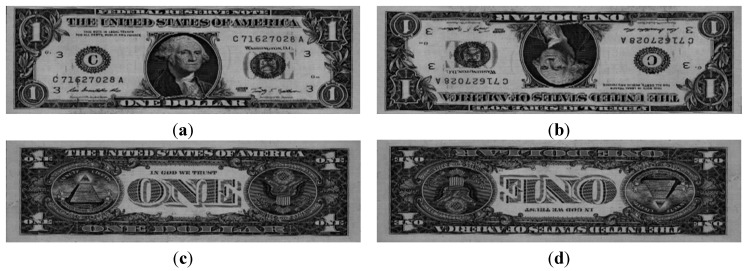
Examples of images: (**a**) Direction A; (**b**) Direction B; (**c**) Direction C; and (**d**) Direction D.

Figure 4 shows example images of a $1 banknote. In this study, the database of 61,240 images was randomly divided into two subsets, Group 1 and Group 2, for training and testing, respectively, as shown in Table 3.

**Table 3:** Number of images in each group and direction.

Category	Group 1	Group 2	Total Number
Direction A	7692	7689	15,381
Direction B	7618	7621	15,239
Direction C	7692	7689	15,381
Direction D	7618	7621	15,239
Total	30,620	30,620	61,240

On the basis of a two-fold cross-validation scheme, the experiments were performed with Groups 1 and 2 from Table 3. First, we used Group 1 for the training process, and Group 2 for testing. Then, the training and testing processes were performed repeatedly, alternating between Group 1 and Group 2.

**Figure 5:**
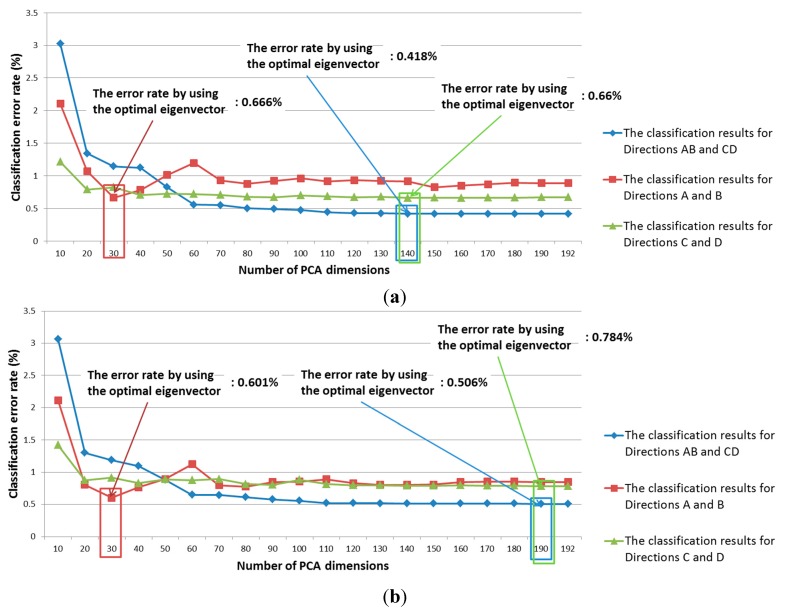
Classification accuracy of PCA according to number of PCA dimensions: (**a**) Group 1; (**b**) Group 2.

In this study, we used PCA to extract features, as explained in Section 2.3. The classification performance is affected by the number of PCA dimensions. Therefore, the optimal PCA dimensionality that produces the best classification accuracy is obtained by experimentation with the training database. This involves measuring the classification accuracy with various numbers of PCA dimensions. Figure 5 shows the experimental classification results for Group 1 and 2 using a Bayesian classifier with various PCA dimensions. The classification error is calculated as the summed value of Type-1 and -2 errors. A Type-1 error means that the obverse side (or forward direction) was incorrectly determined as the reverse side (or backward direction). A Type-2 error means that the reverse side (or backward direction) was incorrectly determined as the obverse side (or forward direction).

In our research, we defined four directions in our study: the obverse side in a forward direction is “Direction A,” the obverse side in a backward direction is “Direction B,” the reverse side in a forward direction is “Direction C,” and the reverse side in a backward direction is “Direction D.” Therefore, “Direction AB” means the obverse side in a forward or backward direction in Figure 5. “Direction CD” means the reverse side in a forward or backward direction.

**Figure 6:**
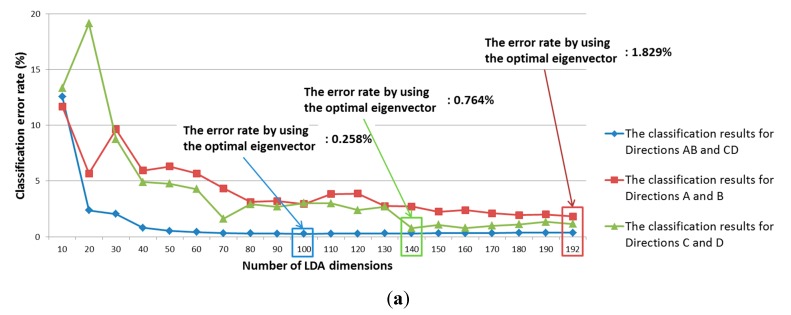

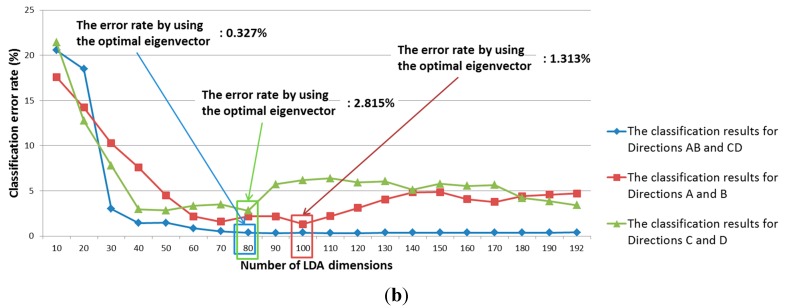
Classification accuracy of LDA according to number of LDA dimensions: (**a**) Group 1; (**b**) Group 2.

For comparison, we also measured the classification accuracy using linear-discriminant analysis (LDA) instead of PCA. The classification results from using the training data are shown in Figure 6.

Table 4 shows the respective classification results from PCA and LDA using the testing data. As shown in Table 4, although LDA outperforms PCA for classifying Direction A and Direction C, the average classification accuracy for the four directions with PCA is higher than it is with LDA.

**Table 4:** Average error rate from two testing sub-databases using LDA and PCA (unit: %).

Method	Direction A	Direction B	Direction C	Direction D	Average Error
LDA	0.241	3.012	1.138	3.708	2.025
PCA	0.689	0.564	1.931	1.352	1.134

**Figure 7:**
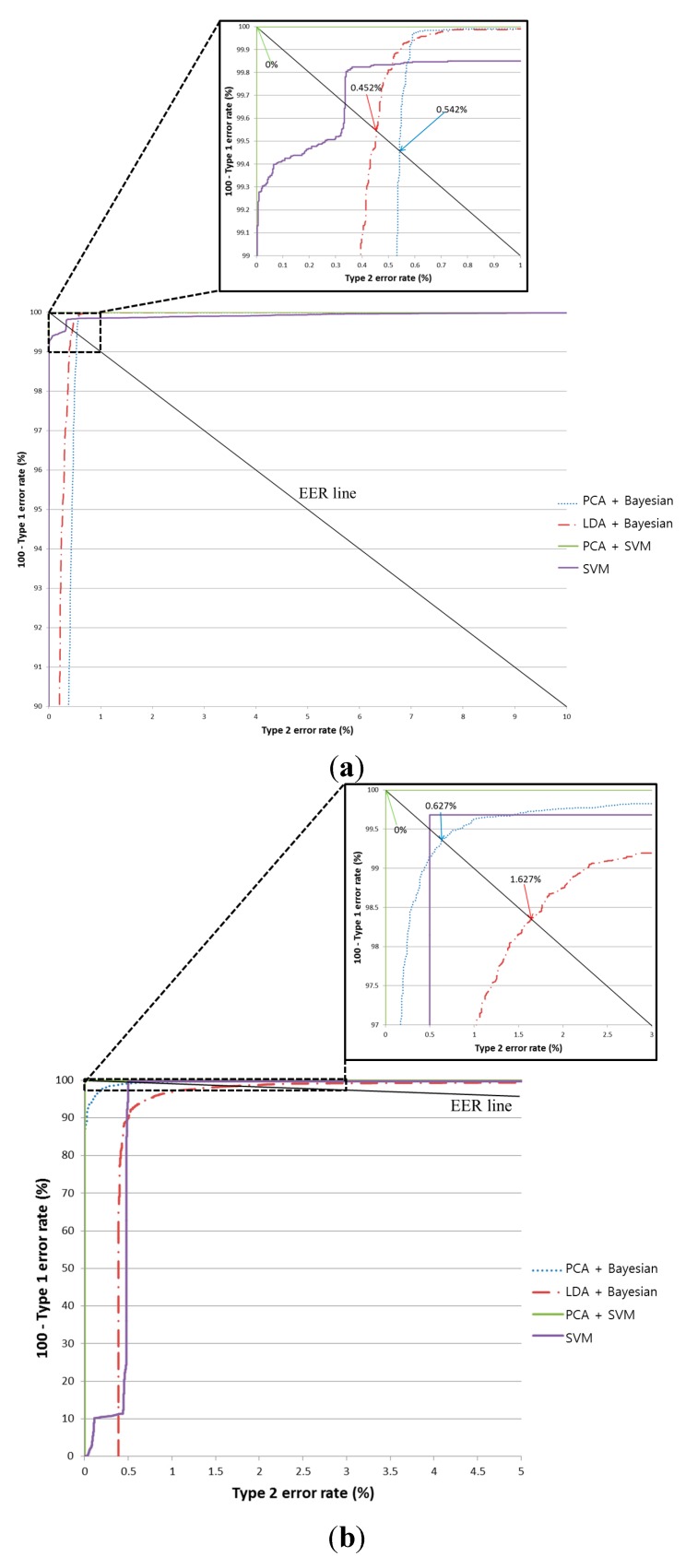

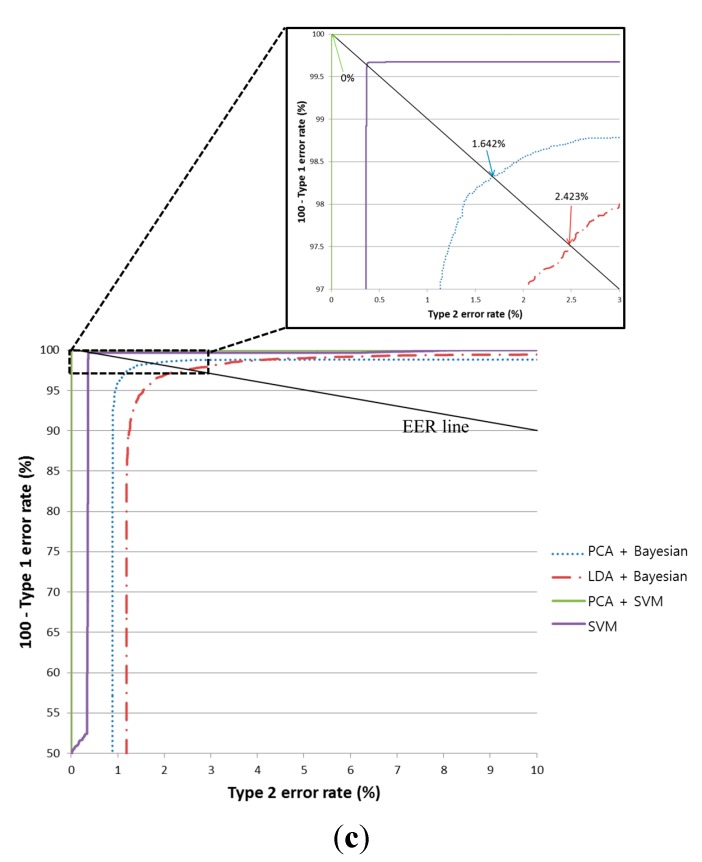
ROC curve from the pre-classification stage using two testing sub-databases (In each figure, PCA+SVM is the proposed method): (**a**) the average classification results for the obverse side (Direction A or B) and the reverse side (Direction C or D); (**b**) the average classification results for Direction A and Direction B; and (**c**) the average classification results for Direction C and Direction D.

To improve the pre-classification performance, we used an SVM classifier instead of a Bayesian classifier for testing by implementing the PCA features with the optimal PCA dimensionality obtained with the training data. In this paper, four SVM kernels were considered: Linear, Polynomial, RBF, and Sigmoid. Using the SVM classifier, the classification accuracy for all four kernels was 100% when using the training database. Therefore, we used the linear kernel in our proposed method because it required processing time is shorter than that of the other kernels. Table 5 and Figure 7 show the results from using the testing database. In Figure 7, equal error rate (EER) means the error rate when the difference between Type-1 and Type-2 errors is minimized.

When using PCA with the SVM classifier, the pre-classification error is 0%. This indicates that no misclassification occurred when our proposed method was applied using the testing database. In addition, the classification accuracy of the SVM is higher than that of the Bayesian classifier using the PCA features. As shown in Table 5 and Figure 7, we compared the accuracy from using Bayesian classifiers with both LDA and PCA. The latter (PCA with Bayesian classifiers) is demonstrably superior to the former (LDA with Bayesian classifiers). As a result, we can confirm that the usability of PCA is higher than LDA. In addition, PCA with an SVM is superior to PCA with Bayesian classifiers, as shown in Table 5 and Figure 7. Hence, we can confirm that the usability of an SVM is higher than that of the Bayesian method. Table 5 and Figure 7 show that PCA with an SVM is superior to merely using an SVM, and we can confirm that the usability of PCA with an SVM is higher than that of an SVM without PCA.

**Table 5:** Average error rates during pre-classification using two testing sub-databases (unit: %).

Method	Direction A	Direction B	Direction C	Direction D	Average Error
LDA + Bayesian	0.241	3.012	1.138	3.708	2.025
PCA + Bayesian	0.689	0.564	1.931	1.352	1.134
SVM	0.419	0.579	0.325	0.441	0.441
PCA + SVM **(proposed method)**	0	0	0	0	0

**Figure 8:**
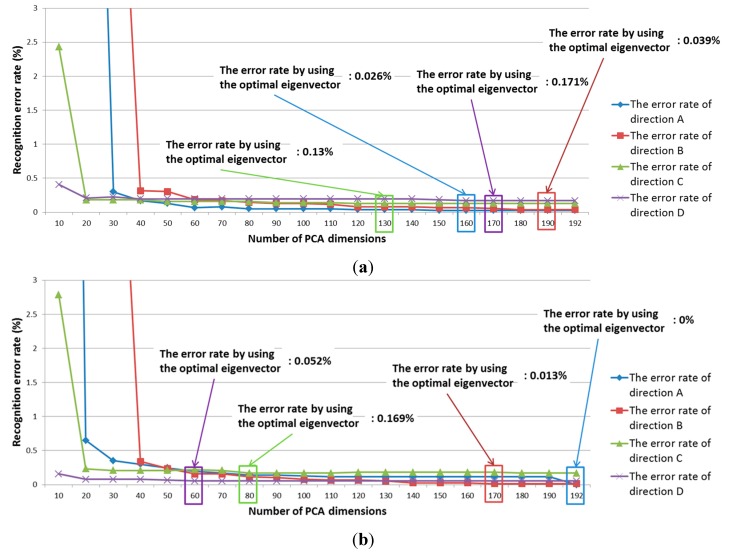
Recognition results for training data using the K-means method: (**a**) Group 1; (**b**) Group 2.

In addition, we performed banknote recognition using a K-means algorithm after the pre-classification of the banknote’s direction. The features for K-means were extracted using PCA. For banknote recognition, the optimal dimensionality of the PCA was experimentally obtained in the same way as it was during the pre-classification step. Thus, the optimal dimensionality was determined to be that which produces the smallest recognition error among various possible numbers of PCA dimensions. Here, the recognition error was calculated at a 100% correct-classification rate. Correct classification means that the input banknote was correctly recognized in its corresponding class—That is, a $1 banknote was correctly recognized as belonging to the $1 class. The recognition results for the training data according to PCA dimensionality are shown in Figure 8. Using the optimal PCA dimensionality for each direction class, the image features were extracted and classified using the K-means method. As shown in Figure 5 and Figure 8, we apply PCA at the three stages such as the pre-classification of obverse (AB) and reverse (CD) sides, that of forward and backward directions (A and B, or C and D), and the banknote recognition by K-means algorithm. The optimal numbers of dimension of the feature vectors after applying PCA are different according to the PCA application for the three stages as shown in Figure 5 and Figure 8.

**Figure 9:**
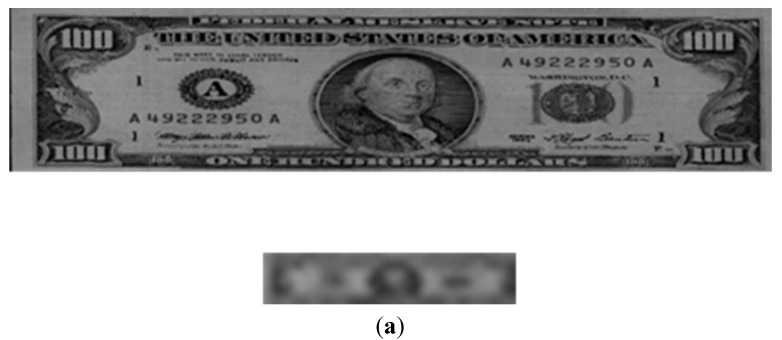

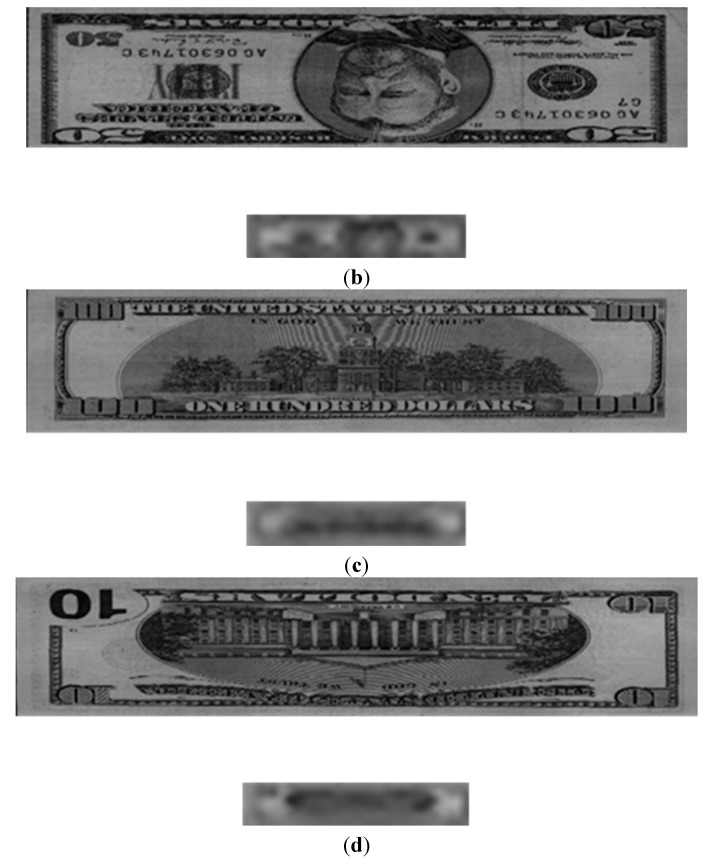
Accurately recognized banknotes and their sub-sampled images: (**a**) Direction A with $100; (**b**) Direction B with recent $50; (**c**) Direction C with recent $100; and (**d**) Direction D with recent $10.

**Figure 10:**
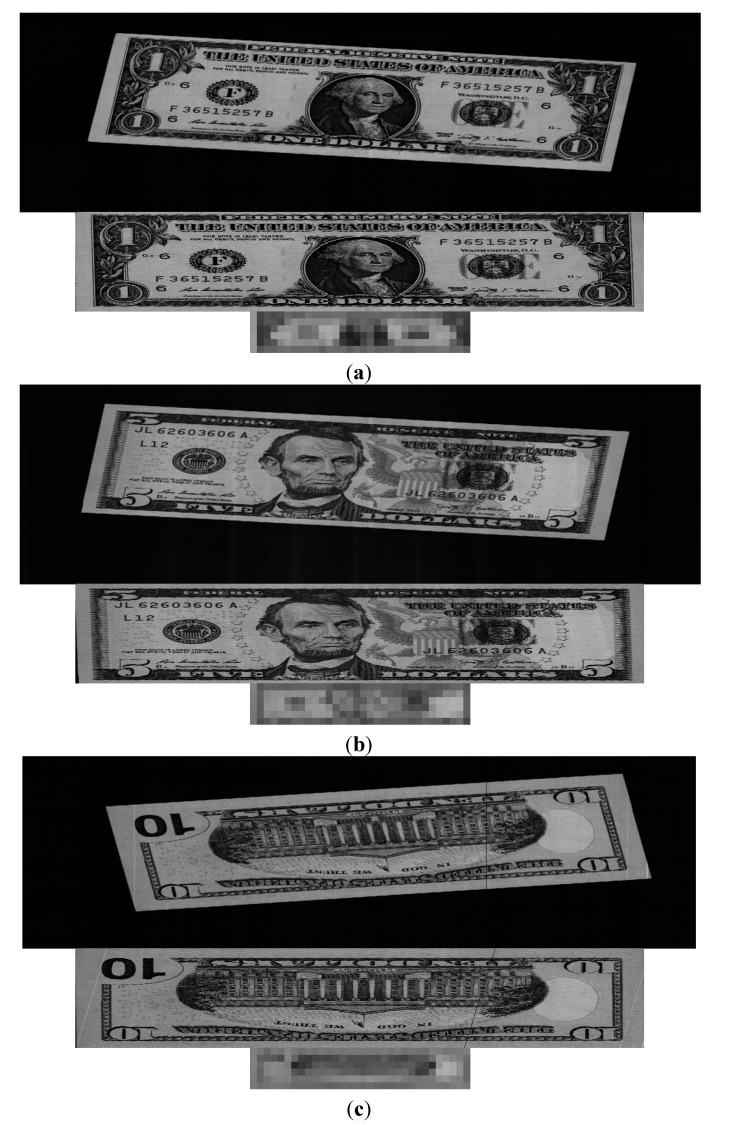
Examples of rotated banknote, the corresponding ROI area, and sub-sampled images. In (**a**)–(**c**), top, middle, and bottom figures are the original input, ROI area, and sub-sampled images, respectively: (**a**) the 1st example; (**b**) the 2nd example; and (**c**) the 3rd example.

Table 6 presents the average banknote-recognition results obtained using K-means with the two testing sub-databases. As shown in Table 6, the error rate for Directions A and B is lower than that of Directions C and D. This is because the reverse side of a USD banknote is less easily discriminated than the obverse, as shown in Figure 9 and Figure 10. On average, the recognition error for the four directions is approximately 0.114%.

Because there is no open source for banknote recognition, it is difficult to compare our method with previous ones. However, because one previous method [12] measured its accuracy with the USD database like our research, we compared the accuracy of [12] to that of our method, as shown in Table 7. In [12], feature extraction is done with QWT and GGD. In our paper, feature extraction is done with PCA. In [12], the classifier used is a BP neural network without pre-classification, while in our paper SVM is used instead.

**Table 6:** Results from performing banknote recognition after pre-classifying the banknote’s direction (unit: %).

Method	Direction A	Direction B	Direction C	Direction D	Average Error
K-means	0.091	0.052	0.176	0.138	0.114

In Table 7, the false-recognition rate refers to the frequency of one banknote image being recognized as an incorrect banknote—e.g., a $10 bill in Direction A recognized as a $10 bill in Direction B, or a $20 bill in Direction A recognized as a $50 bill in Direction A. The reject rate refers to the frequency with which either the type or the class of a banknote is indeterminate. As shown in Table 7, we can confirm that the accuracy of our method is higher than that of previous methods [12] for a greater number of classes and images. Our dataset consists of 61,240 USD banknote images. There was no mistreated banknote included in the dataset. As shown in Table 7, the false recognition rate was 0.114% with the rejection rate of 0% with all the images of 61,240 by our method. Therefore, the correct recognition rate was 99.886%.

**Table 7:** Comparison of the accuracy of the proposed method with previous methods.

Category	Previous Methods [12]	Proposed Method
Number of classes	24	64
Number of USD images	15,000	61,240
False recognition rate (%)	0.12	0.114
Reject rate (%)	0.58	0

Figure 9 illustrates cases where a banknote was correctly recognized with our method. In each pair of images, the lower image is the image sub-sampled at 32 × 6 pixels.

As explained in Section 2.2, the four corner positions of the ROI area on the banknote are detected by corner detection algorithm. Then, the ROI area defined by these four corner positions is segmented from the original input image, and this area is rotated based on the left-upper corner position. From that, the rotation compensated area is obtained as shown in the middle images of Figure 10a–c, respectively. Then, the sub-sampled image of 32 × 6 pixels is acquired based on this area as shown in the bottom images of Figure 10a‒c, respectively. Therefore, the image rotation (the top images of Figure 10a‒c) does not affect the correct acquisition of the ROI area and sub-sampled image, and the consequent accuracy of banknote recognition is not affected by the image rotation.

**Figure 11:**
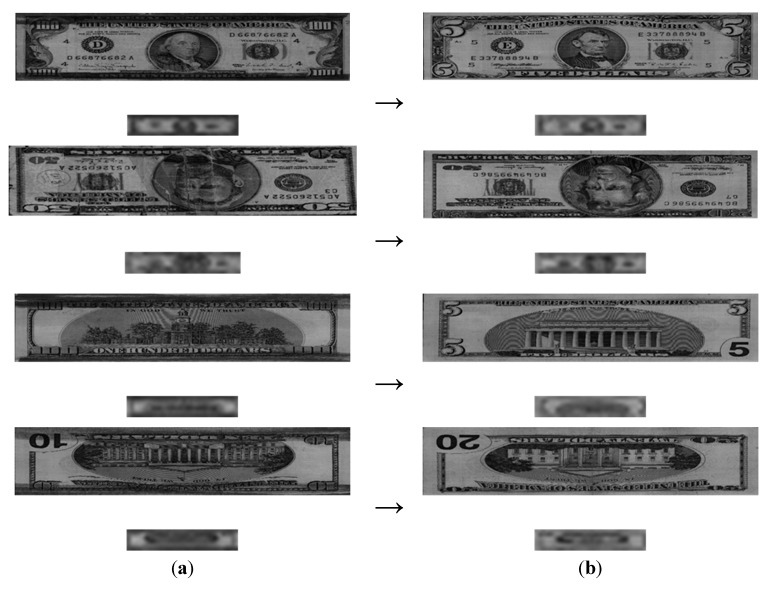
Cases of incorrect banknote recognition: (**a**) input images; (**b**) falsely recognized classes.

Figure 11 illustrates cases where a banknote was incorrectly recognized with our method. In the image pairs, the lower image is the image sub-sampled at 32 × 6 pixels. Each image in the left column was falsely recognized as belonging to the class in the right column. For example, the image in the first row ($100, Direction A) was falsely recognized as belonging the class in the right column ($5, Direction A). As shown in Figure 11, incorrect recognitions occurred when some part of the banknote was damaged (e.g., in the $50-Direction-B image from the second row in the left column) or when a contaminant was present in the upper or lower part of the banknote (e.g., the images in the other rows of the left columns). In particular, as seen in the images from the first, third, and fourth rows of Figure 11, the banknote was falsely recognized because the upper and lower parts of banknote were stained with dark ink. Thus, these cases illustrate a contaminated banknote. In addition, banknotes are falsely recognized when thick vertical lines of discontinuity appear in the mid part of the banknote as a result of being folded, as shown in the images from the second row in Figure 11. This case illustrates a damaged banknote.

In the next experiment, we measured the processing time for our method using a desktop computer (a 3.5 GHz CPU with 8 GB RAM). Most previous research on banknote recognition does not measure the processing time, focusing exclusively on the recognition accuracy instead [9,10,12,13,14,15,16,17]. Therefore, it is difficult to compare the processing time of our method with others. In addition, one extant method measured the processing time during testing but not training [11]. Therefore, we measured only the processing time for our method during testing. As shown in Table 8, our method recognizes a banknote image at approximately 172 images/sec (1000/5.83). The method that measured the processing time [11] required as much as two seconds with a 3 GHz CPU desktop computer and is therefore much slower than our method.

**Table 8:** Processing time of the proposed method on PC (unit: ms).

Image Sub-Sampling	Obverse/Reverse Side Classification		Forward/Backward Direction Classification		K-Means Matching	Total
	Feature Vector Extraction	Side Classification	Feature Vector Extraction	Direction Classification		
2.06	1.27	0.06	1.95	0.04	0.45	5.83

In addition, we measured the processing time on TI DSP. As shown in Table 9, the total processing time is about 15.6 ms and our method recognizes a banknote image at approximately 64 images/sec (1000/15.6) on banknote counting machine.

**Table 9:** Processing time of the proposed method on banknote counting machine (unit: ms).

Image Sub-Sampling	Obverse/Reverse Side Classification		Forward/Backward Direction Classification		K-Means Matching	Total
	Feature Vector Extraction	Side Classification	Feature Vector Extraction	Direction Classification		
4.21	3.24	1.89	3.31	1.91	1.04	15.6

In addition, we measured the total memory usage on banknote counting machine. The total memory usage is about 1.6 MB. It is a total of 734,976 Bytes (1584 × 464) for original input image, 298,152 Bytes (1212 × 246) for the ROI image, 192 Bytes (32 × 6) for the sub-sampled image, 442,368 Bytes (192 × 192 × 3 × 4 (float number type)) for the PCA transform matrix, 2304 Bytes (192 × 3 × 4 (float number type)) for the PCA eigenvalues, 124,716 Bytes for the SVM models, and 49,152 Bytes (192 × 16 (class centers) × 4 (directions of A, B, C, D) × 4 (float number type)) for the K-means algorithm.

## 4. Discussions of Experimental Results

For our research, we obtained the global features from the sub-sampled 32 × 6-pixel image. When comparing the sub-sampled images (of different directions and banknote types) in Figure 3 and Figure 9, we found that each sub-sampled image can be discriminated. As a result, it is clear that the global features (useful for discriminating Directions A–D and classifying the 16 classes, as shown in Figure 1) still remain in the sub-sampled image despite losing the detailed (local) texture patterns. This is confirmed by the accuracy in pre-classification and the recognition rates in Table 5 and Table 7. In addition, using the sub-sampled image enables real-time processing, as shown in Table 8 (172 images/sec (1000/5.83)), with lower memory usage. In general, the misalignment of a banknote often occurs because banknotes are cut inconsistently during production, and this issue is aggravated by variations in the capturing position of the banknote image in banknote-counting machines and ATMs. Misalignment degrades the recognition accuracy, but by using the sub-sampled image, the recognition accuracy is affected less by a misaligned banknote image.

As shown in Figure 3b and Figure 9, the texture components are so blurred in the sub-sampled image that its features are difficult to be extracted by gradient-direction method or Gabor filtering. In addition, because of the limitations to the processing time in a banknote-counting machine or an ATM with low processing power, Gabor filtering—which requires high processing time—is difficult to implement with our method. Therefore, we used the method of extracting features with PCA, and its performance is validated by the results in Table 5, Table 7, and Table 8.

As shown in Table 5 and Figure 7, we compared the accuracy of both LDA and PCA with Bayesian classifiers, and PCA with an SVM for pre-classification. The accuracy from using LDA and PCA with Bayesian classifiers did not reach 100% (the error rates for the former and latter were 2.025% and 1.134%, respectively). In addition, we measured the accuracy of an SVM without PCA, and it did not reach 100%, either, as shown in Table 5 and Figure 7. Based on these results, we are assured that this problem is not simple, and that the usability of PCA with an SVM is higher than in other methods. Because the experiments were done on such a large classes and numbers of USD banknotes compared with previous research (as shown in Table 2), we expect that these results are sufficiently validated.

In our research, we aim at developing an algorithm that can be applied to actual banknote-counting machines and ATMs with low processing power. Therefore, more sophisticated algorithms with higher processing times are unfeasible for these machines due to the limited processing time.

In our research, the final classification of the banknote into 16 classes is also based on PCA features. By using a K-means clustering algorithm, 16 class centers (vectors) are obtained in each Direction A–D, as illustrated in Figure 1. The class mean (center) for each class can be estimated by calculating the average positions (geometric centers) of the data (in each class) in each axis of dimension. However, the accuracy of this method can be affected by the outliers (the error data whose position are far from the center of the class). That is, if the number of the outliers is large, the center of the class is calculated as that close to the outliers, and the incorrect center position can be obtained consequently. However, K-means algorithm is the unsupervised (iterative) learning method which can group the K classes data automatically based on the minimum distance between each sample data and class center. Therefore, its consequent accuracy of determining the correct class center is less affected by the outlier data than that by calculating the geometric center.

With the extracted PCA features from the inputted banknote image, the Euclidean distance between these PCA features and each center (vector) of the 16 classes (which are determined by K-means method) is calculated after pre-classification, and one class center (with the smallest Euclidean distance) is selected as the final class for the banknote (nearest class mean (NCM) method).

In our method, an SVM is used to pre-classify the four classes (Directions A–D) in a hierarchical manner, and a K-means method is used to recognize the banknote from the 16 classes, as illustrated in Figure 1. Because a conventional SVM has been used to discriminate between two classes, and because accurate banknote recognition ultimately requires 16 classes, the SVM was not used for banknote recognition. In order to adopt the SVM for banknote recognition in 16 classes, either a multi-class SVM would be necessary or the conventional SVM would need to be applied repeatedly in a hierarchical way. Both options require too much processing time for use in a banknote-counting machine or an ATM with low processing power. Therefore, we used the K-means method for the banknote recognition in 16 classes. In other words, we performed banknote recognition based on the minimum Euclidean distance with the class center by a K-means clustering method. In addition, because an SVM is usually superior to the K-means method or others for discriminating between two classes (as shown in Table 5), we used the SVM hierarchically to pre-classify the four classes (Direction A–D).

If the ROI area on banknote of 1212 × 246 pixels is used for PCA training, it is difficult to obtain the covariance matrix for PCA training because the size of covariance matrix is so high as 298,152(1212 × 246) × 298,152(1212 × 246) (which requires the memory usage larger than 88 GBs). PCA usually has the functionalities of both dimension reduction and acquisition of optimal features. Therefore, as shown in Figure 7 and Table 5, the average error by PCA + SVM is smaller than that by SVM without PCA. In addition, as shown in Figure 5a, the dimensionality reduction is large (from 192 to 140) in case of the second trial of training when exchanging the training and testing data based on two-fold cross validation. Therefore, although the dimensionality reduction in some cases (Figure 5b) is minimal, the PCA transform with the sub-sampled images is necessary in our research.

Our method can recognize the kind of banknote through the pre-classification of obverse (AB) and reverse (CD) sides, and forward and backward directions (A and B, or C and D) only with the visible light image. Our method cannot identify counterfeits because the detection of counterfeit requires additional information such as magnetic, infrared sensors, *et al*., in addition to the visible light sensor.

## 5. Conclusions

We propose in this paper a novel method for pre-classifying banknotes’ direction for implementation in banknote-recognition systems. The results of our experiments showed that the error rate for the proposed pre-classification method was lower than that of other methods. In addition, the banknote-recognition error rate after pre-classifying the banknote’s direction was as low as 0.114%. However, incorrect recognition occurred when part of the banknote was damaged or when contaminants were present in the upper or lower region of the banknote.

Although banknote images with a limited degree of mistreatment are included in our database, those including tears or hand written notes are not included in our database. As the future work, we would propose to test these images and research a method of enhancing the recognition accuracy with these kinds of poor quality images. In addition, we intend to study an algorithm for rejecting poor-quality banknote images based on a quality measure or the confidence level of the matching score and we plan to apply our method for pre-classifying the banknote’s direction to other currencies, and to compare the accuracy of the method according to the banknote type.

## References

[1] Aoba M., Kikuchi T., Takefuji Y. (2003). Euro banknote recognition system using a three-layered perceptron and RBF networks. IPSJ Trans. Math. Model. Appl..

[2] Takeda F., Omatu S. (1995). A neuro-money recognition using optimized masks by GA. Lect. Notes Comput. Sci..

[3] Kosaka T., Omatu S. Classification of the Italian liras using the LVQ method. Proceedings of IEEE International Conference on Systems, Man, and Cybernetics.

[4] Liu J.F., Liu S.B., Tang X.L. (2003). An algorithm of real-time paper currency recognition. J. Comput. Res. Dev..

[5] Hassanpour H., Farahabadi P.M. (2009). Using hidden markov models for paper currency recognition. Experts Syst. Appl..

[6] García-Lamont F., Cervantes J., López A. (2012). Recognition of Mexican banknotes via their color and texture features. Experts Syst. Appl..

[7] Choi E., Lee J., Yoon J. Feature extraction for banknote classification using wavelet transform. Proceedings of International Conference on Pattern Recognition.

[8] Ahangaryan F.P., Mohammadpour T., Kianisarkaleh A. Persian banknote recognition using wavelet and neural network. Proceedings of the International Conference on Computer Science and Electronics Engineering.

[9] Wu Q., Zhang Y., Ma Z., Wang Z., Jin B. A banknote orientation recognition method with BP network. Proceedings of WRI Global Congress on Intelligent Systems.

[10] Kagehiro T., Nagayoshi H., Sako H. A hierarchical classification method for US bank notes. Proceedings of IAPR Conference on Machine Vision Applications.

[11] Hasanuzzaman F.M., Yang X., Tian Y. Robust and effective component-based banknote recognition by SURF features. Proceedings of the 20th Annual Wireless and Optical Communications Conference.

[12] Gai S., Yang G., Wan M. (2013). Employing quaternion wavelet transform for banknote classification. Neurocomputing.

[13] Ahmadi A., Omatu S., Kosaka T. A PCA based method for improving the reliability of bank note classifier machines. Proceedings of the 3rd International Symposium on Image and Signal Processing and Analysis.

[14] Ahmadi A., Omatu S., Kosaka T. A Study on evaluating and improving the reliability of bank note neuro-classifiers. Proceedings of the SICE Annual Conference.

[15] Ahmadi A., Omatu S., Fujinaka T., Kosaka T. (2004). Improvement of reliability in banknote classification using reject option and local PCA. Inf. Sci..

[16] Omatu S., Yoshioka M., Kosaka Y. Bank note classification using neural networks. Proceedings of IEEE Conference on Emerging Technologies and Factory Automation.

[17] Omatu S., Yoshioka M., Kosaka Y. Reliable banknote classification using neural networks. Proceedings of the 3rd International Conference on Advanced Engineering Computing and Applications in Sciences.

[18] Yeh C.Y., Su W.P., Lee S.J. (2011). Employing Multiple-kernel support vector machines for counterfeit banknote recognition. Appl. Soft Comput..

[19] Bruna A., Farinella G.M., Guarnera G.C., Battiato S. (2013). Forgery detection and value identification of Euro banknotes. Sensors.

[20] Battiato S., Farinella G.M., Bruna A., Guarnera G.C. Counterfeit detection and value recognition of Euro banknotes. Proceedings of International Conference on Computer Vision Theory and Applications.

[21] Hasanuzzaman F.M., Yang X., Tian Y. (2012). Robust and effective component-based banknote recognition for the blind. IEEE Trans. Syst. Man Cybern. Part. C Appl. Rev..

[22] Murphy K.P. (2012). Machine Learning: A Probabilistic Perspective.

[23] Vapnik V.N. (1998). Statistical Learning Theory.

[24] LIBSVM—A Library for Support Vector Machines. http://www.csie.ntu.edu.tw/~cjlin/libsvm.

